# Severe eosinophilic asthma and aspirin-exacerbated respiratory disease associated to eosinophilic gastroenteritis treated with mepolizumab: a case report

**DOI:** 10.1186/s13223-020-00423-3

**Published:** 2020-04-22

**Authors:** C. Caruso, S. Colantuono, D. Pugliese, C. Di Mario, B. Tolusso, E. Gremese, G. Papparella, F. Castrì, A. Gasbarrini, A. Romano, A. Armuzzi

**Affiliations:** 1 Allergy Unit, Fondazione Policlinico Universitario A. Gemelli, IRCCS, Rome, Italy; 2 Department of Translational and Precision Medicine, Sapienza University, Rome, Italy; 3 IBD UNIT Presidio Columbus, Fondazione Policlinico Universitario A. Gemelli IRCCS, Rome, Italy; 4 Division of Rheumatology, Università Cattolica del Sacro Cuore, Rome, Italy; 5 Division of Rheumatology, Fondazione Policlinico Universitario A. Gemelli IRCCS, Rome, Italy; 6 Digestive Endoscopy Unit, Fondazione Policlinico Universitario A. Gemelli IRCCS, Rome, Italy; 7 Polo Scienze della Salute della Donna e del Bambino-Area Anatomia Patologica-Fondazione Policlinico Universitario A. Gemelli IRCCS, Università Cattolica del Sacro Cuore, Rome, Italy; 8 Department of Internal Medicine and Gastroenterology, Fondazione Policlinico A. Gemelli IRCCS, Università Cattolica Sacro Cuore, Rome, Italy; 9 Casa di Cura Quisisana, Rome & Fondazione Mediterranea G.B. Morgagni, Catania, Italy

**Keywords:** AERD, Eosinophilic, Asthma, Gastroenteritis

## Abstract

**Background:**

Mepolizumab (MEP) is the first anti Interleukin (IL)-5 add-on therapy approved for the treatment of severe refractory eosinophilic asthma.

**Case presentation:**

We describe here the case of a 49 years-old woman with Aspirin-exacerbated respiratory disease (AERD), chronic rhinosinusitis, nasal polyposis and eosinophilic gastroenteritis successfully treated with MEP. Several laboratory and clinical items improved during therapy; moreover MEP showed to be useful as steroid sparing agent.

**Conclusions:**

This case supports that the use of mepolizumab can be effective also in other eosinophilic conditions different from asthma and this opens to new therapeutic perspectives.

## Background

Aspirin-exacerbated respiratory disease (AERD) is characterized by chronic eosinophilic nasal polyps, asthma, and airway reactions upon cyclooxygenase (COX) 1 inhibition and affects 1.9% of the European population [1]. Eosinophilic gastroenteritis (EGE) is a rare primary eosinophilic gastrointestinal disorder (EGID), of unknown etiology, characterized by the presence of an intense eosinophilic infiltrate on histopathological examination of the gastric and intestinal mucosa.

The overall prevalence of eosinophilic enteritis is estimated at 5.1/100,000 persons, with peaks of incidence between the third and fifth decade of life [2]. Hypersensitivity response seems to play a key role in pathogenesis of EGE and several patients show association with other conditions such as seasonal allergies, food allergy, asthma, and eczema.

Mepolizumab (MEP) is the first anti Interleukin (IL)-5 add-on therapy approved for the treatment of severe refractory eosinophilic asthma [3]. Eosinophil differentiation, survival, and activation are preferentially regulated by IL-5, a cytokine that binds to the IL-5 receptor (IL-5R), which is located on the surface of eosinophils or basophils and plays a critical role in the pathogenesis and severity of asthma [4]. Recently, one case report of a successfully treatment of both severe asthma and EGE with mepolizumab plus omalizumab (anti-immunoglobulin E monoclonal antibody) has been reported [5].

We describe here the case of a woman suffering from severe eosinophilic asthma and AERD associated to EGE successfully treated with MEP.

## Case presentation

A 49 years-old woman with ten-year recurrent non-bloody watery diarrhea and abdominal pain came to our observation in 2015. No family history of gastrointestinal disorders was detected. A full ileocolonscopy performed 5 years before (no biopsies collected) was normal, leading to a diagnosis of an irritable bowel syndrome, so managed with symptomatic drugs without significant clinical benefit. She had also an history of repeated endoscopic sinus surgery (ESS) because of polyps’ recurrence, aspirin-exacerbated respiratory disease and severe eosinophilic asthma with frequent exacerbations, requiring short courses of oral corticosteroids. Over the years, it appeared that oral steroids induced also a complete relief of gastrointestinal symptoms, with a relapse at withdrawal. Therefore, the patient had repeated steroids exposure, developing dependence and several side-effects.

At admission to our department, she complained watery diarrhea (5–6 bowel movements/day) preceded by abdominal pain. Her vital signs were: SpO2 95% (room air), Temperature 35.8 °C, Heart Rate 100 bpm, Respiratory Rate 18/m, Blood Pressure 108/60 mmHg.

Physical examination was significant only for mild tenderness in the mesogastric area with intact bowel sounds. No organomegaly was found. Visual analog scale (VAS) score for abdominal pain was moderate/severe (60). Besides elevated peripheral blood eosinophil (0.38 × 10^9^/L) laboratory tests were unremarkable. Stool examinations were negative for parasitic, bacteria and clostridium difficile infections. Pulmonary functional tests revealed a Forced Expiratory Volume in the first second (FEV1) of 63% (of predicted value), Asthma Control Test (ACT) score was 13, Sino-Nasal Outcome Test (SNOT-22) was 93, Lund–Mackay (LM) score was 20. Baseline mean OCS dose was 15 mg of prednisone per day. Four OCS requiring asthmatic exacerbations occurred during the previous year.

**Fig. 1:**
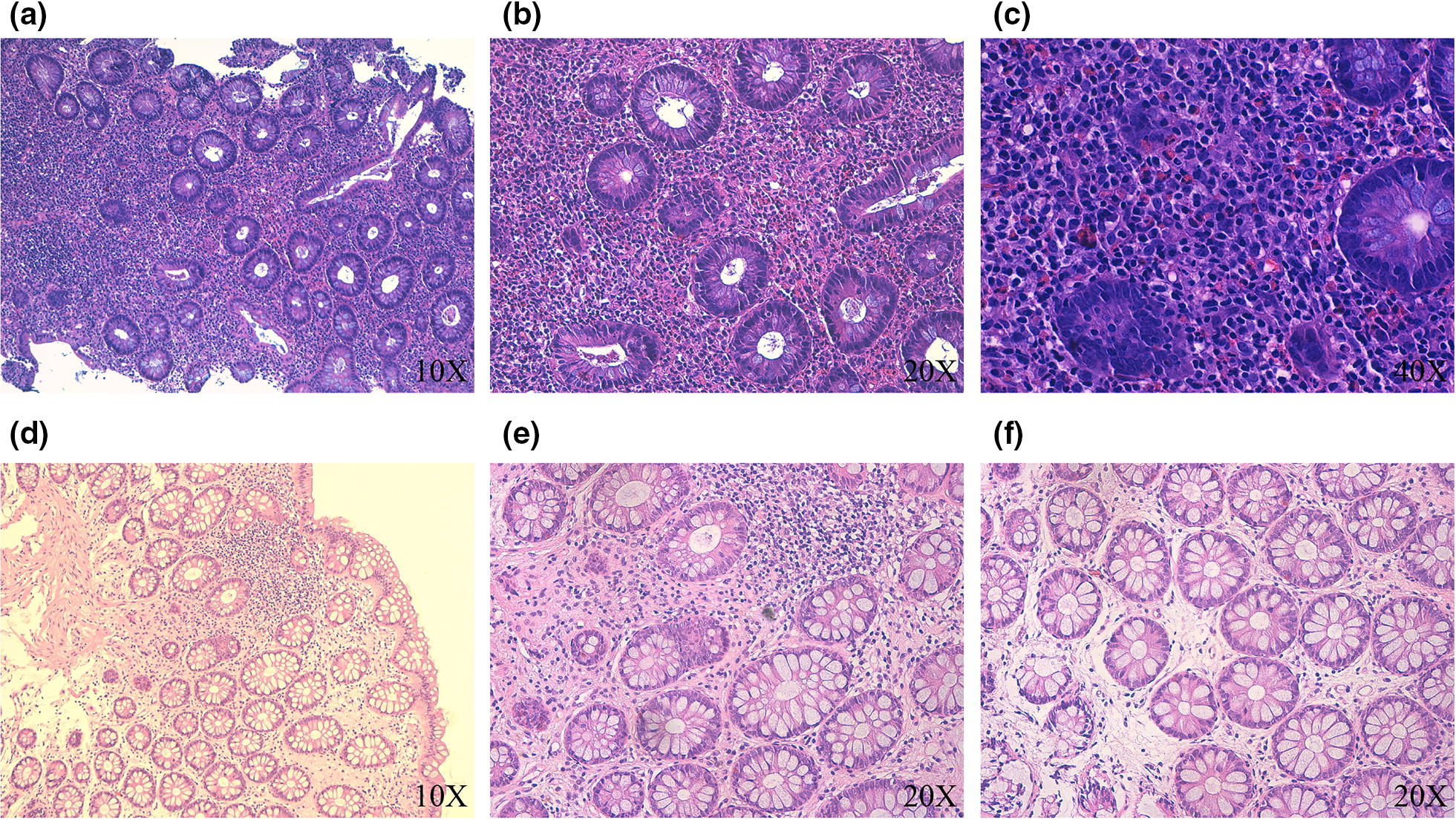
**a** (Hematoxylin and Eosin, ×10): in this picture an important linfoplasmacellular ad granulocytic infiltrate is appreciable in the lamina propria with a predominance of eosinophilis, with an infiltrating pattern sometimes disrupting glandular integrity. **b** (Hematoxylin and Eosin, ×20): a particular of the previous where eosinophils granulocytes surround and infiltrate the glands. As a consequence, glandular structures are depleted of their goblet cells and show reactive hyperchromatic nuclei. **c** (Hematoxylin and Eosin, ×40): this is an atrophic field where the linfomonocytic and eosinophil granulocitic infiltrate disrupt the glandular structures evocating atrophy and reactive changes. **d** (Hematoxylin and Eosin, ×10): this is a picture of the same patient after therapy. You can appreciate the lamina propria devoid of inflammatory infiltrate. The glands are well separated, normoconformed and with a goblet component normorapresented. Just in a focal small field there’s a linfomonocytic infiltrate, where no one can appreciate eosinophilic component. **e** (Hematoxylin and Eosin, ×20): a particular of the previous, where glands are normal and neither a significant eosinophilic infiltrate, nor reactive hyperchromatic changes, nor mucin depletion can be appreciated. **f** (Hematoxylin and Eosin, ×20): as the previous one, glandular mucosal component is conserved and just a focal eosinophilic infiltrate in three different glands is present, but without any specific feature

An ileocolonscopy revealed a normal aspect of the mucosa of each explored segment, except for a small polyp (2 mm) in the rectum, that was removed. Biopsies collected from apparently normal mucosa showed instead an important linfoplasmacellular and granulocytic infiltrate in the lamina propria with a predominance of eosinophils (Fig. 1a, b).

Moreover, no significant findings emerged from gastroscopy, but biopsy specimens, collected in antrum, showed as well as the linfomonocytic and eosinophil granulocytic infiltrate disrupting the glandular structures (Fig. 1c).

According to the immunopathogenetic mechanisms of the diseases and in order to spare oral steroids, in 2017, we started treatment with subcutaneous MEP at the standard dose of 100 mg every 4 weeks, using the severe eosinophilic asthma schedule.

As expected, blood eosinophil count was reduced after the first administration, and became lower and lower during the next 48 weeks (30 cells/mm^3^). After 3 months, patient experienced a significant improvement of sinusitic, respiratory as well as gastrointestinal symptoms. FEV1 and SNOT-22 were evaluated at 6 and 12 month, showing a global improvement (75% and 95%; 51 and 21 respectively). ACT raised to 23. LM score reduced to 8 at month 12. VAS score for abdominal pain reduced to 20 after 3 months so gradually all oral corticosteroids were successfully reduced by patient. After 6 months, patient was in steroid-free clinical remission with a stool frequency of 1–2 movements/day of normal feces (type 3 according to the Bristol stool chart) and a complete resolution of abdominal pain. BMI (Body Mass Index) reduced from 28 to 23 and none asthma exacerbation occurred during the first year of treatment and until the last follow-up evaluation (24 months) [6].

Endoscopies were repeated in order to obtain new biopsy specimens, showing a remission of the disease in term of eosinophilic inflammation, glands where normal and neither reactive hyperchromatic changes, nor mucin depletion can be appreciated (Fig. 1d–f).

**Fig. 2:**
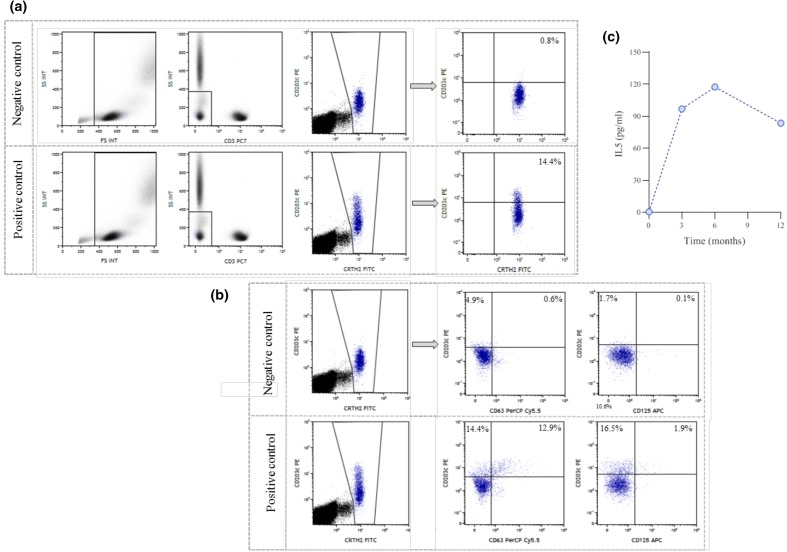
**a**, **b** Flow cytometric analysis of basophils activation. Whole blood was used immediately after collection from the patients. Stimulation of basophils was performed on whole blood according to the instructions of the supplier (Beckman Coulter, France), using the following monoclonal antibodies: CD3-PC7 (clone UCHT1, Beckman Coulter), CRTH2-FITC (clone BM16, Beckman Coulter), CD203c-PE (clone 97A6, Beckman Coulter), CD63-PC5 (clone H5C6, Beckton Dickinson) and CD125-APC (clone 26815, R&D Systems) and the Navios Flow Cytometer (Beckman Coulter). All data generated were analyzed using Kaluza software (Beckman Coulter). **c** Serum levels of IL5 (ELISA; R&D Systems; sensitivity: 0.29 pg/ml) at baseline and after 3, 6 and 12 months of treatment were tested

Basophils activation was evaluated after 12 months of MEP, using the surface molecules CD203c, CD63 and CD125, showing a reduction of activation percentages. Results are shown in Fig. 2a, b [7]. Serum IL-5 levels (R&D Systems; sensitivity: 0.29 pg/ml) were measured at baseline and after 3, 6 and 12 months of treatment and are shown in Fig. 2. Serum IL-5 was non-quantifiable at baseline and showed an increase with peak on 6 months as expected and previously demonstrated by Pouliquen et al. in 2015 (Fig. 2c) [8].

## Discussion

The case of this woman affected by AERD, chronic rhinosinusitis, nasal polyposis and EGE successfully treated with MEP supports recent literature data that show how new biological agents may be useful in eosinophilic conditions other than asthma. Several laboratory and clinical items improved during therapy; moreover MEP showed to be useful as steroid sparing agent. As for AERD, interestingly, at the moment, after 2 years treatment, symptom scores for anosmia (Question 21 from the SNOT-22) and nasal congestion (Question 22 from the SNOT-22) are significantly decreased. Also the response of nasal symptoms and the CT findings obtained with MEP in this patient are remarkable and confirm more recent literature data [9].

## Conclusion

This case suggests that MEP could be effective in the treatment of eosinophilic disorders other than asthma, i.e. EGE and AERD. Obviously long term observations and further investigations are needed to define better the therapeutic management of these diseases, however the description of different cases with different comorbidities could help to define different aspects of therapy in terms of schedule, drug dosage, duration and so on.

## Data Availability

The data used during the current report are available from the corresponding author on reasonable request.

## Abbreviations

MEP: Mepolizumab
AERD: Aspirin-exacerbated respiratory disease
COX: Cyclooxygenase
EGE: Eosinophilic gastroenteritis
EGID: Eosinophilic gastrointestinal disorder
